# Polygenic autoimmune disease risk alleles impacting B cell tolerance act in concert across shared molecular networks in mouse and in humans

**DOI:** 10.3389/fimmu.2022.953439

**Published:** 2022-08-24

**Authors:** Isaac T. W. Harley, Kristen Allison, R. Hal Scofield

**Affiliations:** ^1^Division of Rheumatology, Department of Medicine, University of Colorado School of Medicine, Aurora, CO, United States; ^2^Human Immunology and Immunotherapy Initiative (HI3), Department of Immunology, University of Colorado School of Medicine, Aurora, CO, United States; ^3^Rheumatology Section, Medicine Service, Rocky Mountain Regional Veterans Affairs Medical Center, Aurora, CO, United States; ^4^Department of Medicine, University of Oklahoma Health Sciences Center, Oklahoma City, OK, United States; ^5^Arthritis & Clinical Immunology Program, Oklahoma Medical Research Foundation, Oklahoma City, OK, United States; ^6^Medical/Research Service, US Department of Veterans Affairs Medical Center, Oklahoma City, OK, United States

**Keywords:** systemic lupus erythematosus (SLE), autoimmune type 1 diabetes mellitus (T1D), polygenic, monogenic, genome-wide association study (GWAS), autoimmune disease mouse model, central and peripheral tolerance (anergy), B cell receptor (BCR) signaling pathway

## Abstract

Most B cells produced in the bone marrow have some level of autoreactivity. Despite efforts of central tolerance to eliminate these cells, many escape to periphery, where in healthy individuals, they are rendered functionally non-responsive to restimulation through their antigen receptor *via* a process termed anergy. Broad repertoire autoreactivity may reflect the chances of generating autoreactivity by stochastic use of germline immunoglobulin gene segments or active mechanisms may select autoreactive cells during egress to the naïve peripheral B cell pool. Likewise, it is unclear why in some individuals autoreactive B cell clones become activated and drive pathophysiologic changes in autoimmune diseases. Both of these remain central questions in the study of the immune system(s). In most individuals, autoimmune diseases arise from complex interplay of genetic risk factors and environmental influences. Advances in genome sequencing and increased statistical power from large autoimmune disease cohorts has led to identification of more than 200 autoimmune disease risk loci. It has been observed that autoantibodies are detectable in the serum years to decades prior to the diagnosis of autoimmune disease. Thus, current models hold that genetic defects in the pathways that control autoreactive B cell tolerance set genetic liability thresholds across multiple autoimmune diseases. Despite the fact these seminal concepts were developed in animal (especially murine) models of autoimmune disease, some perceive a disconnect between human risk alleles and those identified in murine models of autoimmune disease. Here, we synthesize the current state of the art in our understanding of human risk alleles in two prototypical autoimmune diseases – systemic lupus erythematosus (SLE) and type 1 diabetes (T1D) along with spontaneous murine disease models. We compare these risk networks to those reported in murine models of these diseases, focusing on pathways relevant to anergy and central tolerance. We highlight some differences between murine and human environmental and genetic factors that may impact autoimmune disease development and expression and may, in turn, explain some of this discrepancy. Finally, we show that there is substantial overlap between the molecular networks that define these disease states across species. Our synthesis and analysis of the current state of the field are consistent with the idea that the same molecular networks are perturbed in murine and human autoimmune disease. Based on these analyses, we anticipate that murine autoimmune disease models will continue to yield novel insights into how best to diagnose, prognose, prevent and treat human autoimmune diseases.

## Introduction: B cell development, autoimmunity and autoimmune pathology

Upwards of 75% of bone marrow produced B cells express B cell antigen receptors (BCRs) that bind self-antigen (1–8). Several mechanisms conspire to remove these autoreactive BCRs from the diverse repertoire needed to provide effective protective humoral immunity without autoimmunity. These mechanisms act both centrally by receptor editing and clonal deletion and peripherally by anergy (7). Central tolerance mechanisms typically remove clones from the wild type repertoire with the most avid interaction with autoantigens. However, peripheral tolerance or anergy is the operative mechanism that silences most autoreactive B cells (3–6). Anergy arises as a consequence of chronic antigen receptor stimulation in the absence of second signals (4, 7, 8). It is defined by non-responsiveness to re-stimulation through the BCR. Importantly, in several B-cell dependent human autoimmune diseases, most individuals with clinically apparent autoimmune disease develop serologically detectable autoantibodies prior to clinical diagnosis (9–13). While we would define B cell dependence as the ability of a B cell depleting therapy to prevent or treat human disease, the inclusion of type 1 diabetes and multiple sclerosis as a B-cell dependent diseases is not universally accepted. However, paired with the clinical efficacy of B-cell targeted therapies either in prevention or treatment of diverse autoimmune pathologies (11, 13–29) these observations implicate dysregulation of central tolerance mechanisms, peripheral tolerance mechanisms or both in the etiopathogenesis of these diseases. Evidence supporting regulatory defects in both central (30–33) and peripheral (31, 32) tolerance mechanisms have been described in numerous human autoimmune pathologies. Central B cell tolerance defects have been described in human SLE (34–36), T1D (37), RA (38, 39) and Sjogren’s Syndrome (40). Peripheral B cell tolerance defects have been described in T1D (41), Autoimmune Thyroid Disease (AITD) (42), SLE (43–46), RA (47–49) and anti-neutrophil cytoplasmic antibody (ANCA)–associated vascluitis (AAV) (50). Current immunologic paradigms hold that immune systems have been selected to balance response to pathogens with damage to self (51–53). If this dominant theoretical framework of immunology is correct, the observation that such high levels of autoreactivity are the norm in some ways challenges our teleology of (auto-)immunity. Indeed, this apparent paradox is perhaps not surprising, as our aim is to reduce a complex system that has evolved to specifically, efficiently and flexibly respond to a universe of molecules with a range of approximately quintillion possibilities (54) to a simple and understandable set of rules.

There are obvious (and non-obvious) differences and drawbacks inherent in extrapolating principles to human pathologies from animal model systems (55). Nevertheless, our understanding of the mechanisms that regulate both central (33) and peripheral B cell tolerance (3, 56, 57)as well as the development of autoreactive B-cell dependent autoimmune pathologies (58–61) has been informed by frameworks developed in murine animal models. Indeed, our current models of the etiopathogenesis of human autoimmune pathology largely consist of a consilience of inductions from both observation and experimentation on living humans, model systems comprised of human tissues/cells and study of murine model systems. However, several have challenged the use of animal models to understand autoimmune pathologies (55). One reason cited for this challenge is that advanced tools for studying human immune responses (62–66) (i.e. CyToF, single cell RNA-sequencing, spectral flow cytometry) now allow more precise definition of human immune responses. Another reason cited for this challenge are high-profile failures in translating findings from animal model of autoimmune disease to humans (67, 68) (some oft cited failures in translation include: oral tolerance with insulin in type 1 diabetes prevention (69), subcutaneous administration of partial agonists to induce antigen-specific T cell tolerance in multiple sclerosis (70–72), the use of interferon gamma (73) and inhibition of TNF-alpha (74, 75) in multiple sclerosis). Importantly, the most often cited high-profile failures in translation have arisen from observations in the EAE (Experimental Autoimmune/Allergic Encephalitis) murine model of multiple sclerosis. Notwithstanding the difference between mice and human beings, challenges in translation are perhaps not surprising, given that clinically defined human phenotypes may well represent congeries of etiopathogenic and pathogenetic mechanisms (76–78). That is, in these diseases each individual actually takes a single path to disease development out of many possible routes. Likewise, each murine model system of autoimmune pathology may well represent a single pathogenetic route to disease development.

Here we synthesize the recent advances in our understanding of the complex genetic basis of two paradigmatic human B-cell dependent autoimmune diseases: Systemic Lupus Erythematosus (SLE) and Type 1 Diabetes Mellitus (T1D). SLE is the prototypical protean multi-system autoimmune disease, whereas type 1 diabetes is the prototypical organ-specific autoimmune disease invariably leading to pancreatic beta-cell destruction. Importantly, both of these disease states have long been modeled with mouse strains that spontaneously develop disease features that closely resemble several of the key phenotypes and pathophysiologies of the human diseases being modeled. Because of the long history of investigation of the cellular and molecular mechanisms of these models, we expect that models of these two diseases are likely to have a more complete list of the genetic contributors and understanding of the relevant cellular and molecular mechanisms leading to murine autoimmune disease.

To address this overlap, we also synthesize what is known regarding the function of putative causal genes across murine models of both systemic autoimmune pathologies (SLE and T1D) and autoreactive B cell tolerance. We discuss several plausible potential explanations for the non-monotonic relationship between currently known human and murine autoimmune risk alleles. Through this analysis, we show that the molecular networks comprised of putative human and murine risk alleles for B-cell dependent autoimmunity and autoimmune pathology substantially overlap. Finally, we propose a framework for steps toward more successful translation of findings from murine model systems to clinical application in humans.

## SLE and T1D: Heritability and epidemiology

In humans both SLE and T1D have heritable component with sibling recurrence risk ratios (lambda S) indicating a substantive genetic contribution (Lambda S SLE = 20, Lambda S T1D = 15) (79). Both are incompletely penetrant, with the monozygotic twin concordance rate estimated to be *at most* 40-50% but likely substantially lower for both diseases (79). Thus, for both of these autoimmune pathologies, non-heritable factors also impact disease development. These non-heritable risk factors are often assumed to represent exposure to one or more environmental triggers. Other stochastic events, such as somatic mutation or particular antigen receptor rearrangement towards a pathologic autoantigen could also plausibly contribute. In SLE the non-heritable component has been estimated to account for ~56% of disease risk (80) and in T1D, this has been estimated at ~34% (81).

In terms of epidemiology, SLE is both more prevalent and more severe in several populations of predominately non-European ancestry than in populations with European ancestry (82). A recent cause of death analysis puts these differences in stark contrast (83). Whereas SLE is the 10th leading cause of death in all female persons aged 15-24 in the US, it is the 5th leading cause of death in African American and Hispanic female persons. Similarly, a recent population-based registry reported approximately 30% mortality within 10 years of diagnosis in Black SLE patients, whereas white SLE patients from the same population exhibited approximately 10% mortality. These differences are likely due to a complex mixture of factors. Potential contributions to these disparities likely include systematic population level differences in access to healthcare and possibly also genetic variants that are exclusive to a particular ancestral group (84, 85). However, population level genetic differences explain only 16% of genetic variability in human populations (86). Therefore, systemic population level differences in access to care may have a greater impact on outcome differences in SLE. A recent report estimates that SLE occurs in US male persons at a rate of 8 to 53 per 100 000 and US female persons at a rate of 84 to 270 per 100 000, depending on the population (87). Importantly, SLE exhibits sexual dimorphism, occurring more commonly in female persons at rate of 9:1 (87). A caveat to the studies referenced above is that they rely on medical record abstraction and administrative data analysis methods that by their nature preclude obtaining sex, gender, race and ethnicity self-identification.

In terms of epidemiology, T1D is reported to be more prevalent in persons who self-identify as non-Hispanic white, followed by non-Hispanic black, Hispanic and other racial/ethnic identities (0.35 to 2.55 per 1 000) with approximately equal prevalence in boys and girls in the US (1.93 per 1 000) (88). T1D incidence increases with age, peaking between 10-14 years of age. Notably, cases with onset < six months of age are not entirely uncommon (89). However, for reasons that remain incompletely clear, the overall incidence of T1D is increasing according to several studies performed in the US (90–92). As a result, based on anticipated demographic shifts, the prevalence is projected to increase from 2.13 per 1 000 in 2010 to 5.20 per 1 000 by 2050 (88). Increasing incidence in recent decades is not unique to type 1 diabetes amongst other autoimmune diseases (93).

When taken together with the observations that different geographies have different rates of autoimmune diseases (94) and autoimmunity (at least the rate of antinuclear antibody seropositivity) has also increased over the same time course (95), these data have been interpreted to strongly imply a changing autoimmunity/autoimmune disease risk environmental exposure has change in recent decades, as the kinetics seem too fast for a genetic explanation.

Several environmental factors have been associated with SLE, including smoking, silica exposure, exogenous sex hormones and infection, especially prior Epstein-Barr virus infection (96, 97). Similarly, in T1D, microbiome, micronutrient, diet, early life metabolism and immune stimuli (infection and vaccination) have been implicated with risk for incident disease (98).

In sum, both SLE and T1D in humans are complex diseases where both genetic and environmental factors contribute both to disease development and disease manifestations.

## Nosology and classification – *Autoimmune* T1D and the heterogeneity of SLE

Both SLE and autoimmune type 1 diabetes pose practical challenges in disease definition, diagnosis and classification that should be considered when evaluating the utility and applicability of any disease model. One cannot evaluate whether a model recapitulates human disease pathogenesis if the definition of disease is unclear.

The particular nomenclature of autoimmune type 1 diabetes may strike the reader as oddly redundant, but it makes the point that type 1 diabetes is a clinical diagnosis. This diagnosis is made in part through typical seropositive autoimmunity to several pancreatic islet expressed proteins (insulin, ZnT8, IA-2, GAD65) (9) in the setting of insulin deficiency. This clinical scenario has been alternately referred to as type 1a diabetes or as immune-mediated type 1 diabetes (99–101). However, a small proportion of individuals clinically diagnosed with type 1 diabetes in large cohort studies have been found to have an alternative etiology for their disease that is non-autoimmune. These individuals commonly have either childhood onset monogenic type 2 diabetes (102) or fulminant onset diabetes with non-autoimmune beta-cell destruction. This latter category of disease has been alternatively referred to as type 1b diabetes, idiopathic type 1 diabetes or nonautoimmune diabetes plus IS (Insulin Sensitivity) (99–101). In some type 1 diabetes cohorts this proportion may be as high as 10% (103). Prior decades of careful phenotyping and molecular characterization has led to description of several subphenotypes of what would have previously considered either type 1 diabetes (young onset, insulin sensitive and autoimmune) or type 2 diabetes (later onset, insulin resistant non-autoimmune). These include latent autoimmune diabetes of adults (LADA), type 1.5 diabetes, ketosis-prone type 2 diabetes and maturity-onset diabetes of the young. See (104) for an excellent review of the nosological challenges of clinical diabetes classification. Our distinction in nomenclature seeks to differentiate monogenic causes of clinical type 1 diabetes with pathologic autoimmunity from monogenic causes of diabetes that clinically resemble autoimmune type 1 diabetes, but arise from non-autoimmune causes. This distinction is clinically important, as management is substantially different (insulin replacement vs. sulfonylureas and other therapies) (105). Indeed, cohorts clinically diagnosed and treated as type 1 diabetics with potential alternative etiologic explanations have been described (106). There is a growing body of literature that using polygenic risk scores (106) and/or sequencing panels of non-autoimmune monogenic risk alleles can help distinguish these two phenotypes. This approach may even be cost effective in select situations (107). Further highlighting the potential for case misclassification in type 1 diabetes cohorts, several recent studies applied type 1 diabetes polygenic risk scores (PRS) to define individuals with clinical type 1 diabetes with low genetic risk (108–110). As expected, these analyses identified rare T1D risk variants in or near genes with well-known effects on immune responses. In addition, these studies identified several rare risk variants in genes with metabolic function or impacts on obesity and no known function in immune responses. Taken together, they suggest that many of the type 1 diabetes cohorts used for GWAS studies likely include a mixture of individuals with autoimmune type 1 diabetes (T1aD) and individuals with non-autoimmune type 1 diabetes (T1bD).

By the same token, SLE is a clinical diagnosis. In order to develop homogeneous patient populations for clinical studies, several iterations of classification criteria have been developed (111–115). The most recent revision was published in 2019 (115). However, most studies of SLE in the past two decades defined SLE cases according to the 1997 revised classification criteria (113). It has been observed that the 1997 criteria lead to 330 possible combinations of clinical manifestations that could satisfy SLE classification (76). Thus, despite being unified by anti-nucleic acid/anti-nucleoprotein autoimmunity (116), human SLE remains a clinically heterogenous disease state. Since particular patients differ in which features of SLE they manifest, attention must be paid to which features of human SLE a particular murine model recapitulates.

## Genetic structure: The usual structure of human autoimmune diseases is polygenic

It is becoming increasingly clear that in most humans who develop autoimmune disease, disease most commonly arises from a complex interplay between many polygenic risk factors and one or more environmental triggers (79). Decreased cost of genotyping and the increasing size of autoimmune disease genetic cohorts has led to a seemingly ever-increasing list of disease risk loci. Indeed, for several common autoimmune diseases, the number of risk genetic loci across the genome now exceeds 200 (117). Each of these loci makes at most a modest contribution to relative risk of disease (odd ratio < 1.2) (117) and most are favored to act by regulating target causal genes (118–120). Together these risk alleles are thought to set a liability threshold that allows the development of autoimmune pathology in certain circumstances. These rules for human autoimmune pathologies appear to generally apply in the case of SLE and T1D with some subtle differences (caveats)?. One notable difference is that of association genetic association with the Major Histocompatibility Complex (MHC)/Human Leukocyte Antigen(HLA) Locus. In T1D, specific HLA alleles are associated with disease. Together, three amino acid variants account for nearly 30% of the phenotypic variance in T1D in European ancestry populations (121). This is similar to the case in RA, where specific HLA alleles have been shown to facilitate binding and presentation of the classic RA autoantigen, citrullinated peptides (122). In SLE, on the other hand, the major contribution to genetic association with the MHC/HLA locus has been mapped to Complement component 4 (*C4A* & *C4B*) gene copy number (123). Both *C4A* and *C4B* are genes that lie within the SLE association interval within the MHC/HLA locus. It has been shown that, in contrast to RA and T1D, the contribution of amino acid sequence variants to the SLE association at the MHC/HLA locus is minimal. HLA is not uninvolved in SLE etiopathogenesis, as there are additional contributions to SLE risk at this complex genetic locus that are attributable to regulation of MHC class II expression (123). However, the bulk of the risk from HLA in SLE arises from regulation of the complement system and not specific MHC alleles (123).

In terms of genetic structure, SLE is most commonly polygenic (117), but numerous monogenic forms of SLE have been described, 51 of which we are aware (124–196). Monogenic SLE presents more commonly with childhood onset and a severe disease phenotype (117, 124–126). It appears that in addition a minority of childhood onset cases, currently estimated at approximately 15% exhibit a probable mix of monogenic and polygenic genetic etiologies (197, 198). Ongoing studies suggest that rare or private mutations also partially contribute to risk in multipatient SLE pedigrees. However, the extent to which such mutations contribute to SLE risk is still being defined (199). To synthesize what is known about polygenic causes of SLE, we applied a previously described approach to published SLE risk variants in the NHGRI-EBI GWAS catalog (117). First, we grouped SLE risk variants listed in the GWAS catalog (200) into loci/regions, then integrated published results the from Open Targets Genetics (201) Locus to Gene (L2G) (202) algorithm. L2G is a machine learning pipeline that predicts a causal gene by integrating several sources of evidence. These sources include distance from causal credible set variants to gene, molecular QTL co-localisation, chromatin interaction data and where applicable variant pathogenicity prediction from the variant effect predictor algorithm. This evidence is then weighted by gold-standard functionally demonstrated causal variants from different GWAS studies. For loci where L2G was able to be confidently annotate a likely causal gene, that gene was included in the molecular network. This list is not comprehensive. Our approach to region definition obscures several known regions with multiple independent genetic effects. Despite this, we find 182 polygenic human SLE risk loci. By applying the L2G automated machine learning pipeline and manual annotation our final list includes 109 loci with assignable putative causal genes within these loci (**Supplementary Table 2A**).

In contradistinction to SLE, only very few (8 of which we are aware – **Supplementary Table 1B**) monogenic causes of autoimmune type 1 diabetes have been described (203–213). Monogenic autoimmune T1D arises in genetic syndromes of polyendocrinopathy. These autoimmune diseases are characterized by autoimmunity that adversely impacts multiple endocrine organs, not merely the pancreas. Only eight monogenic routes to autoimmune diabetes have been described provides a contrast to SLE. This may be in part due to the diffuse, systemic nature of SLE versus the more narrow target organ range of T1D. While SLE exhibits considerable clinical and phenotypic heterogeneity (214) that is unified around anti-nucleic acid/anti-nucleoprotein autoimmunity (116), type 1 diabetes leads to autoimmune pancreatic beta cell destruction. So, it may merely be that in this case there are more opportunities to develop an immune dysregulation syndrome resembling one or more features of SLE, as the manifestations of SLE are both numerous and diverse.

In individuals with T1D, the disease more commonly arises from the aggregate effects of polygenic risk alleles, just as with SLE. Indeed, in the comprehensive review of monogenic autoimmune type 1 diabetes to date reflects the experience of approximately 500 individuals worldwide (203). Thus, monogenic genetic effects or rare genetic effects of large effect size do not likely explain a significant proportion of type 1 diabetes patients and this also appears to be the case in several autoimmune diseases (215). To explore this risk gene network we applied the same approach to define a high confidence causal polygenic risk gene network in human type 1 diabetes. This analysis of type 1 diabetes risk loci from the GWAS catalog yields a list of 131 polygenic human T1D risk loci. The L2G algorithm was able to confidently identify 63 putative causal genes within these loci (**Supplementary Table 2B**). Again, our approach likely obscures the presence of multiple independent signals in a particular region. A recent GWAS meta-analysis of T1D reported that 33% of the independent association signals occurred in loci with multiple independent association signals within the same locus. These independent signals within the same locus might exert their biological effects on disease risk through the same gene. Alternately, these multiple independent signals might exert their biological effects on disease risk through multiple independent genes.

**Figure 1:**
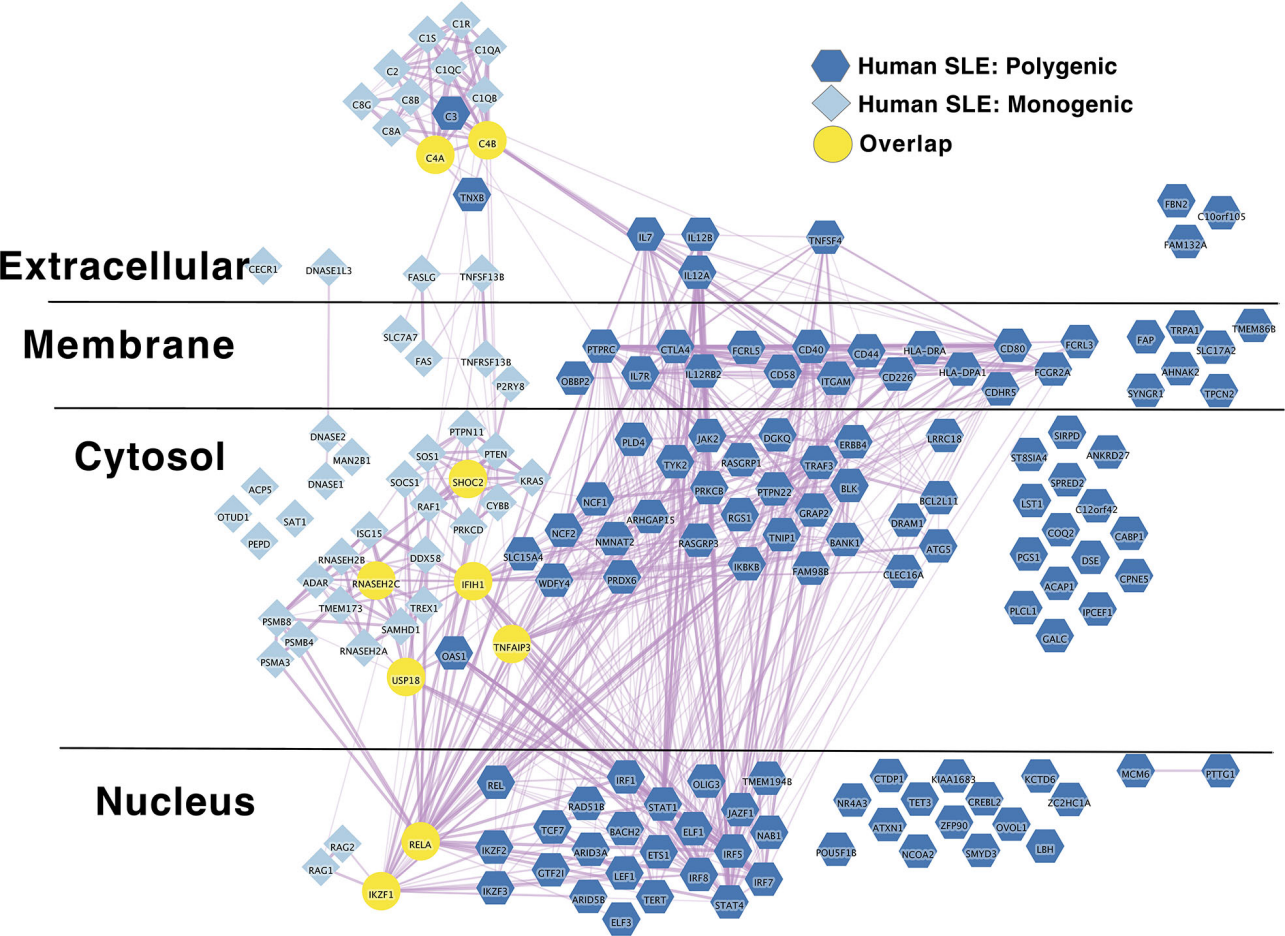
Monogenic and Polygenic human SLE risk gene networks overlap at hub genes. Light blue diamond – Monogenic human SLE genes; dark blue hexagon – Polygenic human SLE genes; Yellow circles – overlapping genes. Downloadable/Interactive network diagram can be found at: https://doi.org/10.18119/N9231T.

**Figure 2:**
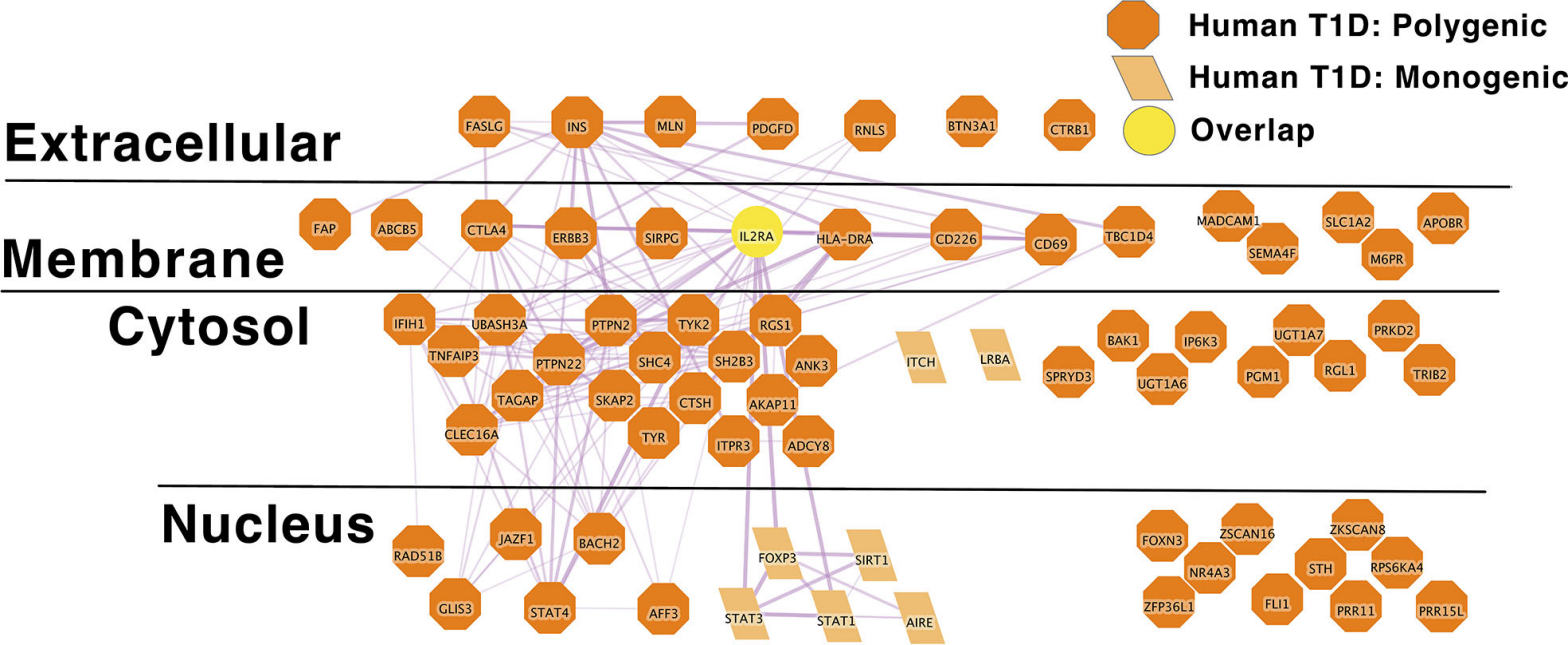
IL2RA is the link between Monogenic and Polygenic human type 1 diabetes risk gene networks; light orange parallelogram– human monogenic autoimmune type 1 diabetes gene; dark orange octagon– human polygenic autoimmune type 1 diabetes gene; Yellow circles – overlapping genes.Downloadable/Interactive network diagram can be found at: https://doi.org/10.18119/N94W34.

*IL2RA* stands out as an algorithmically defined putative causal genes that is also present in the list of monogenic autoimmune type 1 diabetes genes (**Supplementary Table 1B**) as has been observed by others (216). Like SLE (**Figure 1**), the monogenic and polygenic type 1 diabetes risk networks overlap at this hub node (**Figure 2**). This suggests that these hub nodes may be particularly attractive as targets that span disease states based on their central location in both monogenic and polygenic disease molecular networks. In sum, the overlap between polygenic and monogenic disease genetic networks in both human autoimmune Type 1 Diabetes and SLE indicates that the monogenic forms of these diseases perturb the same diseases networks as polygenic disease.

## Beyond polygenic genetic structure: Human autoimmune disease and the omnigenic model

A few general points concerning polygenic genetic structure should be considered. One objection that has been raised to polygenic structure in complex human disease is that sporadic cases are common. Sporadic refers to cases without a known family history of disease. However, statistical genetic models predict that sporadic cases of complex genetic disease will commonly occur even in disease with a polygenic genetic structure (217). Second, the bulk of polygenic risk alleles reported to date in common autoimmune disease only have small effects. In human SLE, as an example, only a handful of common genetic risk factors (four that we know of) impact disease *relative* risk from 2-10-fold (117). Applying knowledge of population prevalence, the genetic factor with the largest effect would change the absolute risk of SLE from approximately 0.1% to 0.4% (117). This kind of polygenic genetic architecture is present in many human phenotypes. This observation prompted the proposal of the Omnigenic model of complex traits (218). In this model, larger effect size variants (>1.1-fold increase in relative risk) operate within core disease pathways. However, thousands of loci with infinitesimally small effect size spanning the entire genome change absolute genetic liability (218). In this model, the entire genome is ultimately involved in disease risk, with each variation outside of the core disease pathway adding a very tiny amount of residual risk. In simple terms, it seems perhaps tautological to state that the whole genome is involved in any given trait, even if only slightly changing the trait. It is worth noting that predictions of this model appear to hold in other complex human genetic traits, such as height (219).

As an aside, the omnigenic model provides a potential explanation for why autoimmune disease genes have not been eliminated *via* natural selection. If most of the hundreds of core risk alleles are inherited independently (low correlation or linkage disequilibrium) and they each have a small effect, then selective pressure would not be expected to be strong in individuals with polygenic autoimmune disease. By way of analogy, being related to someone who wins the lottery does not make winning the lottery more likely for you, unless you buy more lottery tickets. On the other hand, many monogenic disease genes represent either *de novo* mutations or recent founder effects. Therefore, monogenic mutations have not had a very long to be subject to natural selection. These observations when combined with theoretical frameworks describing the balance between host collateral damage from immune responses and microbe clearance (51–53) may also explain the retention of these alleles in the wider gene pool. That is, there are several ways in which immune responses can be balanced to avoid damage to host. Genetic variation that modulates an immune response that is too weak or too strong for one context, may, in another context or in another generation better strike that balance.

If the omnigenic model is correct and thousands of risk loci are involved in determination of common polygenic traits, then sample sizes of > 1 000 000 affected individuals may be needed to develop risk scores that capture enough variants to explain the majority of variation in genetic risk (220). For most autoimmune diseases, these samples exceed the total number of affected individuals living on entire continents. If true, it would make systematically dissecting genetic network interaction with environmental disease triggers so complicated as to be potentially intractable. Our aim is to deconstruct disease processes, in order to improve our ability to diagnose, prognose, prevent and treat autoimmune diseases. Therefore, we must reduce the complexity of the systems we aim to deconstruct. In this way, we can build conceptual models of autoimmune disease development and maintenance that we can actually comprehend.

One approach is murine models. Such models may strike an appropriate balance between over-simplification and a sufficient degree of biological complexity such that core disease relevant cellular and molecular networks are conserved. Thus, findings can be expected to translate to humans. When proper controls and careful attention to potential confounders is observed, mouse models of disease have been very powerful in advancing our understanding of autoimmune pathologies (59).

## Even the lousiest models of autoimmune disease would predict success if considered in context

Having an intermediate model of sufficient biological complexity is likely necessary for many types of causal evidence that allow inference regarding mechanism in cellular and molecular disease networks. In many cases this kind of inference cannot be achieved for either ethical or technical reasons in humans and are inadequately modeled *in vitro*. Many therapies that are promising *in vitro* do not stand up to testing in the more complex biological system that a whole organism *in vivo* represents. One recent example of relevance to autoimmune disease is that of hydroxychloroquine (a mainstay of SLE and Rheumatoid Arthritis therapy (221)) in the treatment of COVID-19. Indeed, hydroxychloroquine robustly inhibited SARS-CoV-2 (and other coronaviruses) *in vitro (* 222), but was shown to be ineffective in prevention of SARS-CoV-2 infection and treatment of COVID-19 in randomized controlled trials in humans (223–226). While it is a moot point now that the high-quality human data exist, an intermediate *in vivo* model system may have been able to predict and understand this therapeutic failure and thereby reprioritized COVID-19 patients for more suitable trials.

Several criticisms of mouse models of human autoimmune pathologies specifically and human disease writ large (with the use of SOD1-deficient mice in Amyotrophic Lateral Sclerosis representing a high-profile model with several issues of phenotypic non-correspondence) have been raised [notably (55, 67, 227, 228)]. See section 5 for our attempt at a comprehensive list of some key variables to consider in modeling human autoimmune disease in mice.

One major criticism that has been raised for why mouse models of human autoimmune disease are ‘lousy’ is failures in translation from experimental autoimmune/allergic encephalomyelitis into successful therapy for multiple sclerosis. However, we would submit that careful attention to both the details of the murine and human pathology and careful reexamination of models in light of the clinical, phenotypic, cellular and molecular features of the human diseases we seek to model would have predicted successful therapeutic targets even in this ‘lousiest’ of autoimmune disease models.

Failed trials of TNF-alpha inhibitors as well as oral and IV tolerance autoantigen-specific tolerance protocols that succeeded in mice, but failed in MS patients are often cited. Incidentally, TNF-alpha inhibition did not merely fail, but was subsequently discovered to be a risk factor for incident demyelination, just as it is a cause of drug-induced lupus. It is worth noting that despite many high-profile therapeutic failures, reassessment of successes, failures and refinement of models have led to several successful novel therapeutic approaches for MS treatment in the interim (68). Subsequently, phenomenally successful trials of B cell-depleting monoclonal antibodies directed against CD20 were performed in MS. In fact, B cells are so important in this autoimmune disease, that B cell depletion using anti-CD20 monoclonal antibodies is now the mainstay of therapy. This is not necessarily a conclusion that would have been reached by solely relying on data from the EAE model (229–234), even though careful experimentation ultimately revealed an important contributory role for B cells once early studies demonstrated the efficacy of anti-CD20 therapies in human MS (235). Subsequent work by many groups has demonstrated that antigen presenting B cells play a central role in the pathogenesis of human MS (236). Building on the principle of the oral tolerance studies in MS, re-enforcing tolerance in formerly anergic B cells remains an active area of investigation (237). More recent data has further advanced our understanding of the role of B cells in MS, as prior Epstein-Barr virus infection (but not other common latent viral infections) was shown to be an independent risk factor for MS development (238), leading commenters to infer that “These findings provide compelling data that implicate EBV as the trigger for the development of MS” (239). These data led to pan-proteome analysis of the auto-specificities of the pathognomonic oligoclonal bands found in the CSF of MS patients. Crossreactivity was shown between a human CNS autoantigen, GlialCAM and the EB viral latency transcription factor EBNA-1 (240). Indeed, as a final attempt to prove etiopathogenesis of EBV in MS – using a modified version of Koch’s postulates, the authors of the latter paper immunized EAE mice and concluded that “EBNA1 immunization aggravates EAE”. In doing so, they have nominated yet another potential therapeutic approach for MS that relies, in part, on the EAE model, the prevention of EB virus infection. In retrospect, the story of the EAE model seems to us more like the typical pattern of advances in science where models are challenged by data and refined so that the model predictions better fit the observed data. Indeed, it now appears that the use of proper controls, challenging murine models with ideas from human data and *vice versa* has an aggregate effect of reducing the influence of potential confounders. In so doing this approach would be expected to lead to a more accurate model autoimmune etiopathogeneis than either approach would have been able to do on its own (60). (many important potential variables are detailed in section 5.)

Thus, despite oft being cited as a model of autoimmune disease with high profile failures in translation, careful attention to the human processes being modeled by the EAE model continues to yield insight into MS pathology. In a similar manner, we expect that careful attention to potential confounders of lupus and T1D models, the use of multiple models and iterative comparison to intermediate human disease phenotypes would be expected to yield important insight into these human autoimmune pathologies.

## Gene networks for murine autoimmune type 1 diabetes, lupus, central and peripheral B cell tolerance overlap

To better understand the relationship between human autoimmune pathology and murine models of autoimmune disease, we compared their respective gene networks. We have focused on making our comparison in long-standing murine disease models of two human autoimmune diseases that are fairly-well characterized in terms of correspondence across spontaneous disease models. For models of both diseases, excellent reviews of the convergent and divergent immunopathogenic bases for disease development between mice and humans have been written and we refer the interested reader to read them: [murine lupus (60, 61, 241, 242): murine type 1 diabetes (243, 244)].

**Figure 3:**
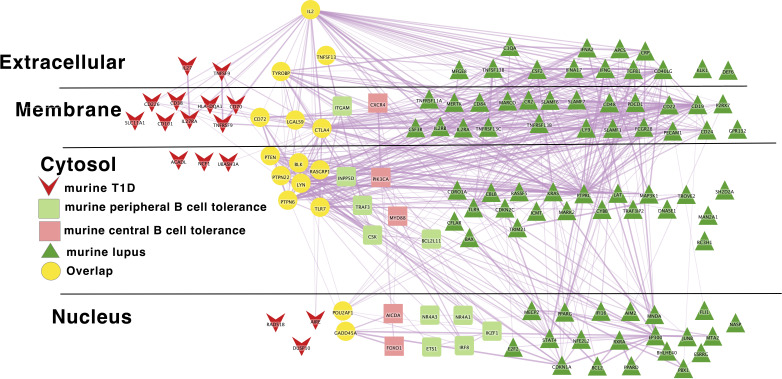
Murine autoimmune diabetes and lupus networks are densely connected to peripheral autoreactive B cell tolerance networks; dark green triangle – murine lupus gene; light green rounded rectangle – murine peripheral B cell tolerance gene; Yellow circles – overlapping genes. Downloadable/Interactive network diagram can be found at: https://doi.org/10.18119/N9161J.

The prevailing model of autoimmune disease risk is that the genetic networks regulating lymphocyte tolerance are core to autoimmune disease and span multiple autoimmunities (56, 57, 245, 246). That is, human genetic risk alleles shared across multiple autoimmune diseases perturb the normal function of lymphocyte self-tolerance networks. To begin both to evaluate this model more systematically and to more fully understand the differences between the murine and human autoimmune disease genetic risk networks, we reviewed the literature and collected lists of putative causal genes in murine models of SLE and type 1 diabetes, as well as genes whose disruption lead to B cell central or peripheral tolerance defects (247–450). Together, each of these sets of genes comprise a molecular network and many of the genes in each network overlap with those in the other networks (**Figure 3**). Taken together, these data point towards an important role of B cell central and peripheral tolerance regulatory networks in murine models of type 1 diabetes and SLE.

## Risk gene networks for murine autoimmune type 1 diabetes, lupus, central and peripheral B cell tolerance overlap with risk gene networks for human SLE and autoimmune type 1 diabetes

**Figure 4:**
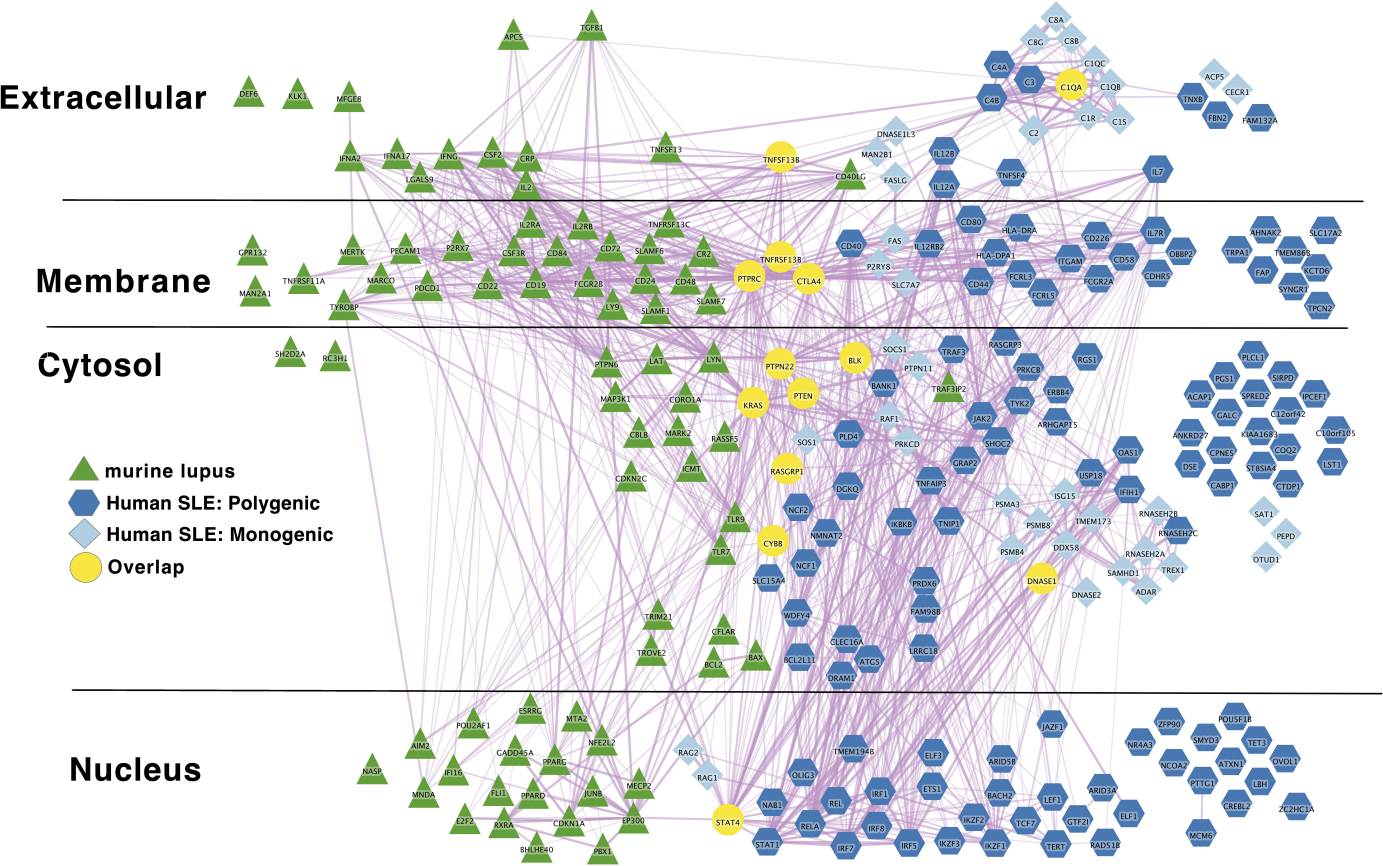
Murine Lupus risk genes connect to Polygenic Human SLE risk genes at the periphery of the core network in a manner similar to the monogenic risk SLE network; Light blue diamond – Monogenic human SLE gene; dark blue hexagon – Polygenic human SLE gene; dark green triangle – murine lupus gene; yellow circles – Overlapping genes. Downloadable/Interactive network diagram can be found at: https://doi.org/10.18119/N9WC8P.

**Figure 5:**
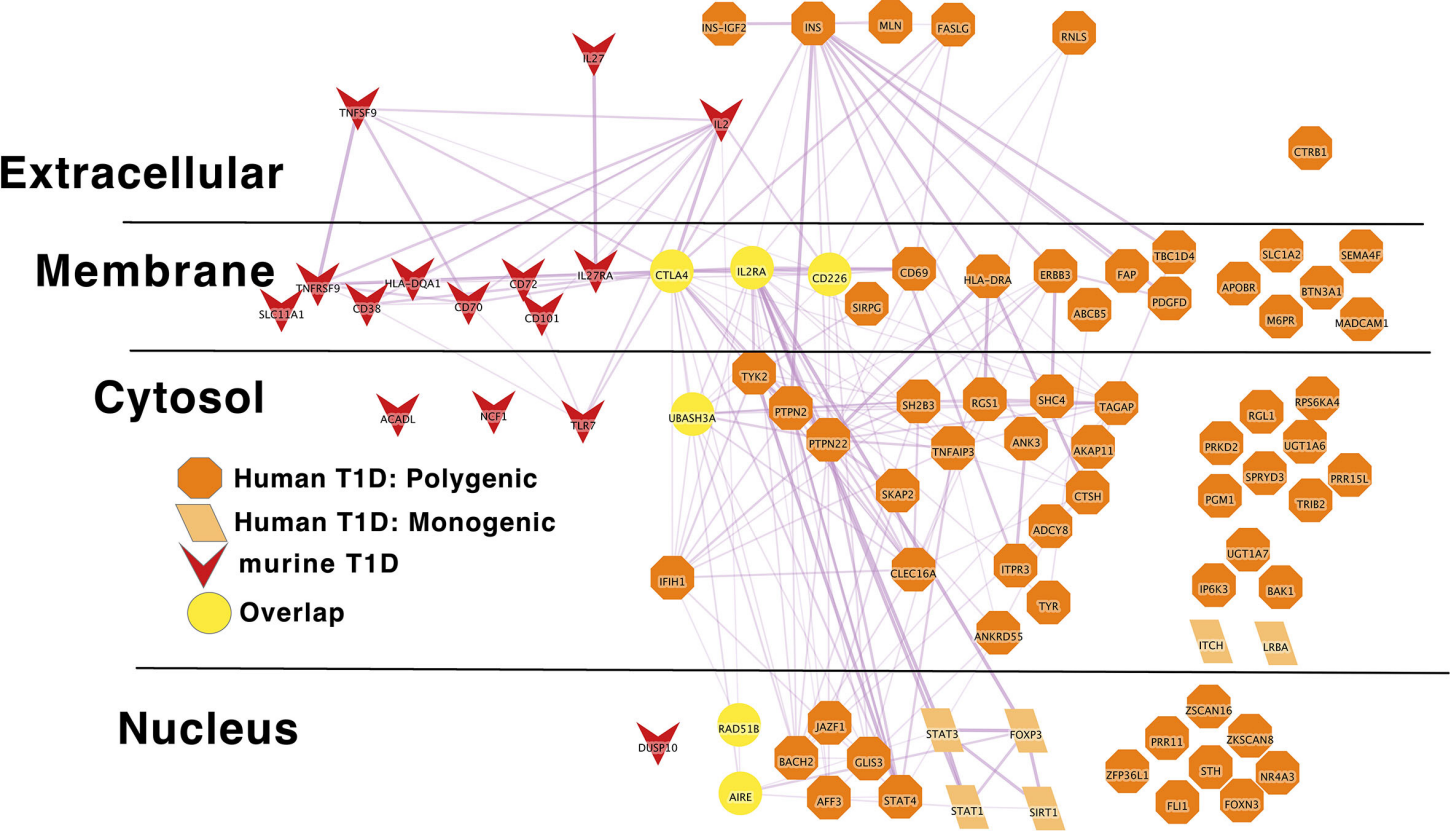
Murine autoimmune diabetes risk genes connect to Polygenic Human T1D risk genes at the periphery of the core network in a manner similar to the monogenic risk T1D network dark red inverted triangle – murine autoimmune type 1 diabetes gene; light orange parallelogram– human monogenic autoimmune type 1 diabetes gene; dark orange octagon– human polygenic autoimmune type 1 diabetes gene. Downloadable/Interactive network diagram can be found at: https://doi.org/10.18119/N9RP6S.

**Figure 6:**
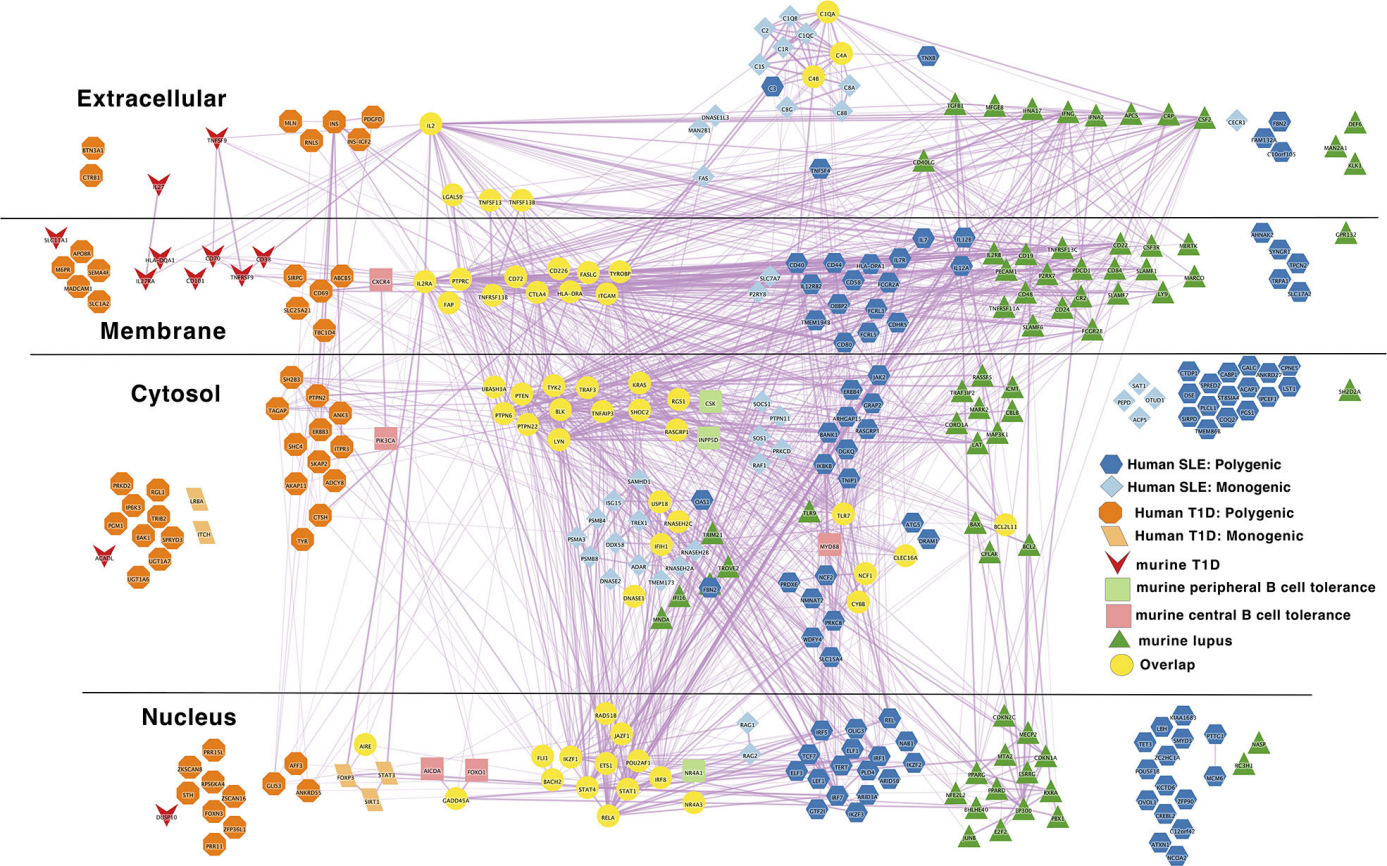
Murine autoimmune disease model genes center around the autoreactive B cell peripheral tolerance network in the middle of combined human autoimmune disease polygenic risk networks. Light blue diamond – Monogenic human SLE gene; dark blue hexagon – Polygenic human SLE gene; dark green triangle – murine lupus gene; light green rounded rectangle – murine peripheral B cell tolerance gene; light red rectangle – murine central B cell tolerance gene; dark red inverted triangle – murine autoimmune type 1 diabetes gene; light orange parallelogram– human monogenic autoimmune type 1 diabetes gene; dark orange octagon– human polygenic autoimmune type 1 diabetes gene; yellow circles – Overlapping genes. Downloadable/Interactive network diagram can be found at: https://doi.org/10.18119/N9MW3G.

To understand how autoimmune disease gene networks overlap, we merged the murine and human risk gene networks for SLE and T1D in several ways. Our goal was to evaluate whether the published studies support the prevailing model – that the genes regulating tolerance induction and escape of autoreactive B cells are central to the risk gene network of these seropositive autoimmune diseases. First, we combined risk genes from monogenic human SLE (**Supplemental Table 1A**), polygenic human SLE (**Supplemental Table 2A**) and murine Lupus genes (**Supplemental Table 3**) into a single network (**Figure 4**). Second, we combined gene from monogenic human T1D (**Supplemental Table 1B**), polygenic human T1D (**Supplemental Table 2B**) and murine autoimmune diabetes (**Supplemental Table 3**) genes into a single network (**Figure 5**). Finally, we combined both of the disease-specific networks (from **Figures 4**, **5**) along with both B cell central (**Supplemental Table 3**) and peripheral tolerance (**Supplemental Table 3**) gene networks into a single network (**Figure 6**). Strikingly each of these gene sets formed a distinct protein-protein interaction network with greater overlap than expected by chance (**Table 1**). Further, the human monogenic and polygenic and murine genetic networks overlap 16-fold to 63-fold more than would be expected by chance (**Table 2**). Likewise, these networks overlap with one another or the overall B cell tolerance and murine disease networks between 15-fold and 86-fold more often than expected by chance (**Table 3**).

**Table 1:** Network characteristics.

Network	#nodes^a^	#edges^b^	degree^c^	clustering^d^	exp. Edges^e^	P^f^
*Monogenic SLE*	54	169	6	0.65	33	1.0E-16
*Polygenic SLE*	127	497	8	0.44	107	1.0E-16
*Monogenic T1D*	8	12	3	0.64	3	2.8E-05
*Polygenic T1D*	70	140	4	0.37	22	1.0E-16
*murine lupus*	92	523	11	0.58	111	1.0E-16
*murine T1D*	20	31	3	0.58	3	1.0E-16
*peripheral tolerance^g^*	22	63	6	0.58	8	1.0E-16
*central tolerance^g^*	7	7	2	0.24	1	6.7E-04

Network characteristics for each string protein-protein interaction network reveals a highly connected disease network in each gene list.

^a^#nodes indicates the number of genes in the network. ^b^#edges indicates the number of pairwise predicted protein-protein interactions according to the default settings in the string database (http://www.string-db.org) (451). ^c^Degree indicates average node degree. Per the string database manual: “The average node degree is a number of how many interactions (at the score threshold) that a protein have on the average in the network”. ^d^Clustering indicates the average clustering coefficient. Per the string database manual: “The clustering coefficient is a measure of how connected the nodes in the network are. Highly connected networks have high values”. ^e^Exp. Edges indicates “The expected number of edges gives how many edges is to be expected if the nodes were to be selected at random.”. ^f^P indicates the P value for enrichment of this protein-protein interaction network. “A small PPI enrichment p-value indicate that the nodes are not random and that the observed number of edges is significant.” Note: the minimum enrichment p-value reported by string is 1E-16.^g^peripheral tolerance and central tolerance indicate networks of genes implicated in peripheral and central B cell tolerance.

**Table 2:** Disease Network Overlap.

	Disease	Exp. Overlaps^a^	Fold O-R^b^	P^c^
*Human Polygenic: Monogenic Overlap*	SLE	0.35	26	6.8E-11
	T1D	0.03	35	2.8E-02
*Combined Human: Murine Overlap*	SLE	0.82	16	1.6E-12
	T1D	0.08	63	1.2E-08

Overlap of disease networks supporting **Figures 1**, **2** (Human Polygenic: Monogenic Overlap) and **Figures 4**, **5** (Combined Human: Murine Overlap). ^a^Exp. Overlaps indicate the number of expected overlapping nodes. Assuming similar length lists were randomly selected from the genome (unassociated). ^b^Fold O-R indicates the fold over-representation compared to expectation. ^c^P indicates p-value for hypergeometric distribution assuming independence of the two networks.

**Table 3:** Overlaps of disease networks supporting **Figures 3** (Murine T1D, Lupus, Peripheral and Central tolerance) and **Figure 6** (all 8 networks combined).

Network	Overlaps in Figure 3			Overlaps in Figure 6		
	Exp. Overlaps^a^	Fold O-R^b^	P^c^	Exp. Overlaps^a^	Fold O-R^b^	P^c^
*Monogenic SLE*	X	X	X	0.92	18	1.5E-17
*Polygenic SLE*	X	X	X	2.17	15	1.5E-28
*Monogenic T1D*	X	X	X	0.14	22	2.6E-04
*Polygenic T1D*	X	X	X	1.19	16	4.3E-18
*murine lupus*	0.58	26	1.9E-17	1.57	16	1.9E-23
*murine T1D*	0.13	32	6.8E-06	0.34	26	1.6E-11
*peripheral^d^*	0.14	86	1.5E-21	0.38	51	2.2E-31
*central^d^*	0.04	45	8.2E-04	0.12	17	5.8E-03

Overlap of disease networks supporting **Figures 3** (Murine T1D, Lupus, Peripheral and Central tolerance) and **Figure 6** (all 8 networks combined). ^a^Exp. Overlaps indicate the number of expected overlapping nodes. Assuming similar length lists were randomly selected from the genome (unassociated). ^b^Fold O-R indicates the fold over-representation compared to expectation. ^c^P indicates p-value for hypergeometric distribution assuming independence of the two networks. ^d^peripheral and central indicate networks of genes implicated in peripheral and central B cell tolerance. As a negative control, comparison was made to the L2G predicted causal genes in a large GWAS of osteoarthritis (452) and type 2 diabetes (453). In both cases, overlap was substantially less than in the table above. A single putative causal gene out of 19 for osteoarthritis overlapped with the network in **Figure 6**. This corresponds to 3-fold overrepresentation with P-value of 0.27. 17 putative causal gene out of 343 for type 2 diabetes overlapped with the network in **Figure 6**. This corresponds to 2.9-fold overrepresentation with P-value of 9E-5. Of note, the overlapping genes were enriched for genes within apoptosis and cellular proliferation pathways. As these core cellular processes impact both the genesis of autoimmune pathology and insulin resistance, this degree of overlap is perhaps not surprising.

OA network: https://version-11-5.string-db.org/cgi/network?networkId=bWV0Pd2gEYYx.

DM2 network: https://version-11-5.string-db.org/cgi/network?networkId=boNoFGYSyFUn.

Overall, this analysis reveals a densely interconnected core autoimmunity gene network centered around genes that regulate B cell peripheral tolerance. This observation provides some degree of support for the prevailing model in the field, that the genes regulating tolerance induction and escape of autoreactive B cells are central to the risk gene network of these seropositive autoimmune diseases. Intermixed within this core are the murine type 1 diabetes and lupus gene networks. While this approach has utility in providing a high-level overview of autoimmune disease risk regulatory networks, it does have some drawbacks. In each particular network, there are several putative causal genes that are not well connected to the central network. Certainly, it is possible that these genes have yet to be discovered function in the genesis of autoimmunity. However, there are other potential explanations for lack of connection to this central network. In some cases, these may represent misattribution of causality. For example, while the L2G algorithm nominated *PTTG1* as a putative causal gene for SLE, we have previously shown that altered function of the microRNA, *MIR146A*, likely better explains the observed association with SLE at this locus (454). Alternately, these genes may impact lupus function in a way that has not yet been represented in the molecular networks of the STRING database. For example, recent work has established *DNASE1L3* as casual for SLE. First, non-synonymous coding changes in *DNASE1L3* explain the bulk of the genetic association with SLE near the *PXK* locus (455). Second, germline mutations in this gene have been described as a monogenic route to lupus (198, 456–458). Third, titers of autoantibodies against this enzyme correlate with disease flare in patients with lupus nephritis (459). Fourth, functional studies implicate the function of this secreted, extracellular DNAse in digesting the nucleic acids present in autoantigenic debris from dying cells (460–462). Thus, while the role of *DNASE1L3* in SLE risk is becoming abundantly clear, the STRING database (451) has not yet codified this new understanding. At the same time, there may be other information missing from the gene network as we have defined it. At this same locus, *DNASE1L3-PXK*, an additional contribution to genetic association with SLE is seen (455). This additional association is due to variation near *PXK*, a phox-homology kinase implicated in B-cell receptor endocytosis (463). There is evidence for a potential role of PXK in modulating B-cell receptor signaling and generating autoreactivity. However, the automated algorithmic approach that we used did not place *PXK* within the polygenic SLE risk network. While this approach provides a useful overview of the interrelationships between gene networks, by its nature, it also provides an incomplete picture of disease risk due to incomplete information.

On a more granular level, these analyses revealed overlapping networks between monogenic and polygenic SLE. This overlap was between complement, cytosolic nucleic acid sensors, Ikaros and NF-kB pathways (**Figure 1**). In terms of monogenic and polygenic autoimmune type 1 diabetes, not surprisingly, there is limited overlap (**Figure 2**). However, there is still more than expected by chance. This includes a preponderance of key transcriptional regulators (*STAT1*, *STAT3*, *FOXP3*, *AIRE*) that are central regulators of T lymphocyte development in monogenic T1D. Close inspection of these networks shows that they do not overlap at *AIRE*. This lack of overlap highlights one of the drawbacks of the automated, algorithmic approach to putative causal gene definition. A rare variation in *AIRE*, rs74203920, was recently reported in a large GWAS of human autoimmune type 1 diabetes (464). This non-synonymous variation results in an amino acid change that is predicted to be deleterious. It has a minor allele frequency of ~2% in individuals with European continental ancestry in the 1000 Genomes project. Further, using Bayesian statistical approaches, the authors report a posterior probability of association > 99% (464). There are examples of non-synonymous coding changes in GWAS genes whose biological effects on disease risk may be through modulation of gene expression (465). However, it seems most parsimonious to conclude that *AIRE* is, in fact, the likely causal gene at this T1D risk locus. That our approach using L2G did not identify this particular variant and it therefore did not overlap with the monogenic T1D risk network highlights one of the drawbacks of this approach in terms of misattrubtion. It further suggests that our overlaps are more likely to represent a lower bound on the overlap between the true disease risk networks than an upper bound.

Turning to the network that combnines murine lupus, murine T1D and murine B cell tolerance gene networks, we also find substantial overlap. This overlap occurs within several pathways: IL2 (IL2), BCR signaling (BLK, Lyn etc.), tolerance response to nucleic acid (CD72, TLR7), tolerance to self-nucleic acid and control of viral infection. These overlaps serve as unifying pathways in these models of autoimmune pathology (**Figure 3**). Overlap of murine and human lupus occurs at B-cell signaling hubs involving BAFF, APRIL and B cell antigen receptor signaling. Of note, despite its central importance in SLE etiopathogenesis (117), TLR7 is absent from the human disease networks, though its signaling intermediates remain. Likewise, LYN is absent from the human disease networks despite its identification as a likely causal gene for SLE in GWAS follow-up studies. (**Figure 4**) Thus, our analysis likely underestimates the true extent of overlap between these various gene networks. Similar to Lupus, type 1 diabetes in mouse and humans is unified by T-cell tolerance regulators (CTLA4, IL2RA, CD226, AIRE, etc.) (**Figure 5**). Finally, peripheral B cell tolerance is the most over-represented compared to no association when looking at the unified network of all these states of pathologic autoimmunity (**Figure 6**). The substantial overlap between these different networks is consistent with a prominent role of particular environmental drivers in specifying the target organ focus of autoimmunity.

One question that arises is whether these associations represent an increase over what would be expected by chance. Indeed, overlap between the gene networks in type 2 diabetes (453) and osteoarthritis (452) are much less with these non-autoimmune traits than any of the autoimmune pathology networks (**Table 3**). Another question is how to address cell type specificity of these networks. One might assume that these gene networks only operate in concert within specific cell types. PTPN22 may serve as a counterexample to this – a recent review highlighted evidence for six independent mechanisms of the PTPN22R620W variant each operating in different cellular lineages (466). It may be that some autoimmune disease risk alleles do act in a cell type and cellular context-specific way. However, for many complex human traits, the genetic structure predicted by the omnigenic model appears to be the case. That is, hundreds to thousands of genetic variants of (mostly) very small effect size act in aggregate to set a genetic liability threshold. The central nodes in these disease gene networks have the largest effect size and therefore likely a lower statistical power requirement to demonstrate association. Thus, like many pharmacotherapies (467), it may well be that these core disease genes have multiple mechanisms through which they modulate disease risk. Hence, they are centrally located and have outsized effect sizes. Certainly, BLK, Lyn and the BAFF family genes in these networks could be argued to have effects selective to the B cell lineage. However, both BAFF (468) and Lyn (469) have well described actions outside of B cells. Likewise, BLK exhibits high expression in human plasmacytoid dendritic cells (470, 471) and the most strongly associated eQTL variants are within human fibroblasts. Both of these cell lineages are independent from B cells and have direct relevance to SLE etiopathogenesis. A role for these three genes acting to increase SLE risk within B cells is certainly more parsimonious. Alternately, it has been argued that several of the polygenic risk variants for human type 1 diabetes exhibit opposite action in effector and regulatory T cells (472). That is, several risk variants increase the likelihood of activation in effector T cells and simultaneously increase the likelihood of inhibition in regulatory T cells. Thus, even with specific cellular mechanisms, the risk alleles of the strongest effect size may be the most likely to have multiple mechanisms whereby they alter disease risk. Cogent arguments can be made for the cellular specificity of gene networks acting within a disease state. However, much work remains to be done to convincingly demonstrate cell-type specificity of genetic effects, over against disease risk networks that span and exert their effects within multiple cellular lineages.

## Potential explanations for gaps in translation

What are the explanations for challenges in translatability of autoimmune disease mouse models?

We have discussed spontaneous, induced and humanized murine autoimmune disease models above in general terms. Here we focus on key potential differences that in our estimation are likely to affect several spontaneous models of lupus, such as those derived from the NZB/NZW F1 (BW) mice and the NOD mouse model of type 1 diabetes.

### Recombinant inbred mice/Polygenic disease in Humans vs. Monogenic disease in mice

The use of recombinant inbred mice more closely resembles consanguinity that is seen more commonly the parents of individuals with childhood onset autosomal recessive disease. In this way, these murine models may offer more opportunities to develop monogenic mutations and sub-strain differences can profoundly alter physiology (473). One example sticks out in particular. The most commonly used lab mouse strain, C57BL6/J, developed a loss of function mutation in *Nnt*, the gene encoding for the nicotinamide nucleotide transhydrogenase (473). This mutant *Nnt* diverges from another commonly used lab mouse strain C57BL6/NJ. Unfortunately, *Nnt* mutation inadvertently serves as a model of familial glucocorticoid deficiency, which has been described in mice and humans who have mutant NNT (474). This could conceivably confound interpretation of results obtained using models that have not controlled for this mutation in lupus in particular, where glucocorticoids are a mainstay of therapy. As another example, a body of literature describing functions previously attributed to caspase-1 are in fact due caspase-11 deficiency due to inadvertent gene-targeting leading to generation of caspase-1/caspase-11 double knockout mice (475).

### Genetic & evolutionary divergence of both host and microbiota

Sixty-five million years of evolutionary history seems like a long time. Certainly, it is long enough to develop changes in how genes respond to the environment. As a stark example, Gout is a disease of higher primates. It is one of the most common forms of inflammatory arthritis and is estimated to affect 1 in 200 people worldwide. Gout occurs when uric acid levels are too high and uric acid crystals precipitate out of the serum, driving acute and chronic inflammation. Gout is thought to have arisen ~ twenty-two million years ago when one of a series of loss of function mutations in uricase (which converts uric acid to the much more water-soluble allantoin) and URAT1 and important renal uric acid transporter. As this system non-redundantly regulates blood pressure, it stands to reason that changes across similarly complex immune networks could have also developed differences in some critical regulatory genes. Indeed, many immune phenotypes that diverge between mice and humans have been described (476). Two select examples of gene to phenotype non-correspondence include MyD88 and STAT5B. MyD88 deficiency leads to early life susceptibility to only pyogenic infections in humans whereas it leads to long lasting susceptibility to a broad array of infections in mice (477). STAT5B deficiency leads to different phenotypes in terms of Treg generation, IL2R signaling and *in vivo* T cell effector function in mice as compared with humans (478).

### Environmental enrichment

While humans are housed in varied circumstances, housing of mice is somewhat uniform. Environmental enrichment (EE) makes mouse housing more “fun” and leads to reductions in a variety of depressive/anxious behaviors and indicators of stress response in mice (479). At the same time, there is evidence that EE substantively impacts the antitumor response of NK cells and immunotherapy treated anti-cancer T cells (480). Thus, differences in the monotony and variety of environment may be a factor that alters immune system responses and could impact autoimmune disease pathways.

### Thermoneutral housing

When given the option, mice, like humans tend to inhabit places with comfortable ambient temperature or change their environment to maintain their own core temperature in the thermoneutral zone. Humans do this by wearing clothes, whereas mice tend to fill their burrows with bedding and insulation. Observation of mice in the wild indicates that during their light cycle, mice tend to maintain a thermoneutral zone of 30-32 degrees Celsius. For historical reasons and for the comfort of clothed humans, most mouse facilities house mice at room temperature 19-25 degrees Celsius. Thus, mice are subjected to chronic “cold stress” which carries with it attendant increased sympathetic nervous system/beta-adrenergic tone and changes in whole organism metabolism and physiology (481). Removal of this cold stress through thermoneutral housing has been demonstrated to impact several immune phenotypes, including notably, induction of oral tolerance (482–485). Further there is growing evidence that the parasympathetic nervous system impacts autoimmune disease. For example, vagal nerve (parasympathetic) (486) stimulation has led to improvement of systemic inflammatory parameters in short-term trials (487, 488).

### Circadian rhythms

Mice are typically handled in the vivarium during daylight hours, a period during which they commonly sleep in the wild. Several autoimmune diseases are associated with sleep disturbance (489) due to incompletely clear mechanisms. Indeed, less than 7 hours of sleep is associated with the onset of human SLE in longitudinal cohort studies (490). Further, several reports indicate that systematically sleep deprived NZB/NZWF (1) mice develop increased lupus activity (491, 492). Thus, differences in circadian cycles may be an additional factor to consider when modeling human autoimmune pathologies in mice.

### Microbiota/pet store mice

Our immune system gene networks have subject to selective pressure for the sixty-five million years since divergence from mice. At the same time, the mutualistic relationship with our microbiota has been under pressure from our immune system and *vice versa*. This may be another important meta-genomic divergence that leads to non-correspondence of murine models of human disease (59). Following our reductionist tendencies, the character and make up of mouse microbiota is being intensively defined and simplified as specific-pathogen-free facilities are increasingly used (493, 494). Normalizing the microbiome to one that more closely resembles wild mice leads to several substantial changes in immune response (495–498). Thus, colonization with comparatively non-immunogenic microbiota may be yet another factor that needs to be accounted for when modeling human autoimmune disease in mice.

### Humans (usually) already have disease: Early disease therapy vs. established disease therapy

Most therapies given to people with autoimmune disorders are usually administered to counter a matured, often chronic disease. While prevention trials are underway in several human autoimmune diseases (221), many therapies employed in mouse models are preventive in nature. That is, intervention occurs prior to the onset of disease.

### Mice are not free to eat what they want (but they can usually eat as much as they want)

Many lab rodent diets contain substantial proportions of alfalfa meal (499, 500). Alfalfa sprout consumption was long ago associated with incident lupus-like disease in higher primates and attributed to the presence of canavanine, a non-canonical arginine-related amino acid (501). Subsequent studies have also found epidemiological evidence of association with lupus (502), to the point that a commonly used Lupus patient education website recommends avoidance of alfalfa sprouts (503). Curiously, anti-cyclic citrullinated peptide antibodies (against peptides with the non-canonical arginine related amino acid citrulline) are commonly seen in individuals with rheumatoid arthritis as well as those with clinical features of both SLE and RA (504). Recent work has also implicated peptide processing that leads to hybrid-insulin peptide formation, generating a neoepitope as etiologic in type 1 Diabetes (505). Protein dietary and metabolic changes could theoretically alter the generation of neoepitopes in alfalfa fed mice and more broadly appear to have an important role in the genesis of several autoimmune pathologies.

### Humans are free

Established disease in humans almost always means confounders – behavior, medications, adherence, understanding, communication, health literacy, numerical literacy, risk perception and risk calculus [COVID-19 pandemic as a global example (506)], to name a few. There is a situation when established disease in humans tends to go along with fewer confounders – early life. However, ethical and practical issues usually prevent trials in children for diseases that also develop in adults. Maybe it isn’t that mice are simple, but that humans are just too complicated?

### Mice are not free and cannot access sunlight

Most research animal facilities, have strict policies against taking mice out of the viviarium for a walk in the sun. This likely lowers the risk for the skin manifestations of lupus, which are importantly mediated by UV. While the artificial environment of the vivarium can be addressed artificially with transient UV exposure (507), vitamin D is also an independent protective factor for lupus flares and the development of several autoimmune disease (508–511).

### Mice have fur

The absence of extensive hair follicles, dermal and epidermal layers that are twice as thick and the absence of a specialized muscle layer (*Panniculus carnosus*) all distinguish human from murine skin (512–514). If histological differences do not pose a sufficient challenge in modeling human skin pathologies in mice, it has been observed that only ~30% of the top skin-expressed genes overlap between mouse and human skin (515). Taken together, these differences pose several problems in modeling SLE, as autoimmune response in the skin is the first disease manifestation in many affected humans.

### Mice are not naturally susceptible to infection by EBV

In addition to implication in MS (discussed above), EBV infection in humans is associated with SLE. There are mechanistic links implicating molecular mimicry by EBNA-1 (516) and substantial enrichment of EBNA-2, the latency transcription factor, at GWAS loci for SLE and other autoimmune diseases (516). There are also examples of allele specific binding of EB viral transcription factors to causal risk alleles. How might this confound translatability of murine model data? The closest gammaherpes virus to EBV that infects mice is murine gamma-herpesvirus 68. While murine gamma-herpesvirus 68 does infect mice, it lacks several features of EBV (517). If one of those divergent features omits a critical step in the EBV-dependent development of autoimmune disease, then this divergence would impact our ability to model autoimmune disease development in a way that parallels what is suspected to occur in humans.

In this section we point out some differences to consider when interpreting murine model data in light of human autoimmune pathology. There are several features of humans that make modeling an inherently error-prone process. These complicating features are in addition to the potential intractability of understanding gene X environment interactions, if the omnigenic model proves true. Despite these drawbacks, murine models of autoimmune diseases have advanced our understanding of the gene networks that regulate autoimmune pathologies. At the same time, efforts at translation require both careful attention to potential confounders and continual reexamination of our models in light of the clinical, phenotypic, cellular and molecular features of the human diseases we seek to model.

## Implications and a potential path towards translation

Simply put, the need for improved understanding and more diverse and less toxic therapeutic options for SLE and Type 1 diabetes is dire. The discrepant severity of SLE outcomes between populations simply cannot be accepted in a just society. To the extent that our lack of understanding contributes to this discrepancy, it needs to be corrected. In a similar manner, Type 1 diabetes disproportionately afflicts some of the most vulnerable members of our society with a burden of chronic disease and a concomitant burden of co-morbidity and mortality. Despite life-saving advances in therapy in the prior decades, the incidence of this disease is rising. So, we must better understand its genesis in order to more effectively intervene.

We need to understand disease mechanisms and define causal genetic immunophenotypes in humans. For this understanding to be certain regarding causal relationships, parallel understanding of mechanism in model systems is required for effective trial design. Mice have proven to be excellent sacrificial companions on our collective journey of disease deconstruction for both SLE and T1D. They have facilitated perturbations of genes and environmental triggers, allowing assessment of the impacts on murine intermediate immune cellular and molecular phenotypes and correlates of pathology. It continues to be prudent to advance therapies that can prove efficacy in these model systems along the path toward clinical application. However, careful attention to the details of both the model system and the disease processes being modeled is necessary to fully evaluate both therapeutic candidate successes and failures. Nearly 90% of trialed pharmaco-therapeutic candidates do not advance to the FDA approval (518). These rates are better for biologics than for small molecules at each stage of drug development, possibly due to the more specifically targeted nature of biologic therapies versus small molecules (519, 520). This failure is despite the best efforts of many who are employed by pharmaceutical companies. Our ability to fully understand these incredibly complex biological systems remains incomplete. Thus, it is perhaps not surprising that there have been several high-profile failures to develop autoimmune disease therapy.

How best to evaluate therapeutic leads for autoimmune diseases? Our proposed approach follows. Cellular/molecular phenotypes and pathological correlates of disease would need to be ameliorated by candidate therapeutic leads in murine systems to a reasonable degree of certainty in terms of causality. At the same time parallel approaches could be validated in human *in vitro* (cell lines), *ex vivo* (primary cells) or *in vivo* (hu-mice) reductionist model systems and shown to return the cellular/molecular phenotypes and pathologic correlates move to a healthier status with any therapeutic lead. Therapies that pass this bar could be trialed in first in human trials after primate evaluation or if repurposing (if already FDA approved), moved directly to phase 3 trials. Human trials based on the cellular, molecular and pathologic frameworks derived from model systems would need to include assessment of correlates of the postulated mechanism. Additionally, evaluation of any competing mechanisms would assist post-hoc evaluation of whether a given trial represented a true trial of therapy. Indeed, two recent (the first two since the 1950s) FDA-approved therapies for SLE, belimumab (anti-BAFF) and anifrolumab (anti-IFNAR1), both took approaches similar to the approach that we lay out. Following identification of antigen-presentation by B cells (521–528) as key in the genesis of murine autoimmune type 1 diabetes there is now a focus on B cell tolerance pathways in human T1D (41, 529–533). Further characterization of the role of B cell tolerance (534) and efforts to manipulate pathogenic autoantigen-reactive B cells in type 1 diabetes promise (530) to bring therapeutic successes in this disease, where T cells have long been the subject of focus. Our analysis highlights a potential role for autoreactive B cell tolerance in the development of multiple autoimmune pathologies. In doing so, it adds to a growing body of work that supports viewing seropositive autoimmunity as an endophenotype of multiple autoimmune diseases (535–541). As our efforts to more broadly understand autoimmune disease polygenic genetic risk network impacts on B cell function advance, we anticipate that murine disease models will continue to be critically important to furthering understanding of autoimmune diseases and advancing the goal of improved outcomes for patients.

## Author contributions

IH conceived of the article, carried out the analyses, drafted and revised the manuscript. KA contributed to data analysis and interpretation and critically revised the manuscript. RS contributed to data analysis, interpretation and critically revised the manuscript All authors contributed to the article and approved the submitted version.

## Funding

IH receives funding from the Rheumatology Research Foundation in the form of a Scientist Development Award. He is also partly funded by the Pfizer Global Grants Foundation Rheumatology program #51849703, but those funds did not support his work on this project. RS receives funding from NIH R01DE029303, VA I01 CX001877 and VA I01 BX001451.

## Acknowledgments

The Genotype-Tissue Expression (GTEx) Project was supported by the Common Fund of the Office of the Director of the National Institutes of Health, and by NCI, NHGRI, NHLBI, NIDA, NIMH, and NINDS. The data used to support the claim that BLK eQTLs occur in fibroblasts were obtained from the GTEx Portal http://www.gtexportal.org/ on 2022-05-10. https://www.gtexportal.org/home/gene/BLK. The BioGPS resource (470), human primary cell atlas (471) was used to identify non B-cell expression of *BLK*. http://ds.biogps.org/?dataset=BDS_00013&gene=640. [Accessed 2022-05-10].

## Conflict of interest 

IH is partly funded by the Pfizer Global Grants Foundation Rheumatology program #51849703, but those funds did not support his work on this project.

The remaining authors declare that the research was conducted in the absence of any commercial or financial relationships that could be construed as a potential conflict of interest.

## Publisher’s note

All claims expressed in this article are solely those of the authors and do not necessarily represent those of their affiliated organizations, or those of the publisher, the editors and the reviewers. Any product that may be evaluated in this article, or claim that may be made by its manufacturer, is not guaranteed or endorsed by the publisher.

## Author disclaimer

The contents of this manuscript do not represent the views of the U.S. Department of Veterans Affairs or the United States Government.

## Supplementary material

Supplementary Table 1A Monogenic Routes to Human Lupus – Gene(s) refers to the gene or genes that when mutated has been reported to lead to lupus or a lupus-like phenotype. Gene name refers to the HGNC (HUGO Gene Nomenclature Committee [https://www.genenames.org/]) official full name for that gene. Locus refers to chromosome and cytoband for that gene. Protein refers to the common protein name for a particular gene. Inheritance indicates the mode of inheritance (if reported) according to the abbreviations at the end of the table. Pathway refers to the reported pathway disrupted by the mutation. Phenotype refers to the phenotype observed, whether part of another defined genetic syndrome, such as Noonan syndrome or whether part of bona fide SLE or another lupus-like phenotype. Reference refers to numbered reference in the bibliography of this publication. PMID or link refers to the pubmed.gov identifier (PMID) for the publication or publications establishing the gene as a monogenic route to lupus or a review of several publications.

Supplementary Table 1B Monogenic Routes to Human autoimmune Type 1 Diabetes – Gene(s) refers to the gene or genes that when mutated has been reported to lead to lupus or a lupus-like phenotype. Gene name refers to the HGNC (HUGO Gene Nomenclature Committee [https://www.genenames.org/]) official full name for that gene. Locus refers to chromosome and cytoband for that gene. Protein refers to the common protein name for a particular gene. Inheritance indicates the mode of inheritance (if reported) according to the abbreviations at the end of the table. Pathway refers to the reported pathway disrupted by the mutation. Phenotype refers to the phenotype observed according to online mendelian mutation in man (OMIM) [https://omim.org/]. Reference refers to numbered reference in the bibliography of this publication. PMID or link refers to the pubmed.gov identifier (PMID) for the publication or publications establishing the gene as a monogenic route to type 1 diabetes or a review of several publications.

Supplementary Table 2A Human SLE Polygenic risk loci from GWAS catalog and putative causal gene(s) as identified by OpenTargetsGenetics L2G pipeline – Variant & Risk allele: the genetic variant with smallest reported P-value for association with SLE by GWAS and the corresponding risk allele as reported by the EBI/NHGRI GWAS catalog [https://www.ebi.ac.uk/gwas/]. P-value: the p-value reported for that variant. P-value annotation: commentary on the P-value reported for that variant as reported in the EBI GWAS catalog (i.e. association in a specific population, conditional logistic regression based on covariates, etc.) RAF – risk allele frequency, if reported in the EBI GWAS catalog. OR – reported odds ratio for the risk allele as reported in the EBI GWAS catalog. Beta – effect size or natural logarithm of the odds ratio. CI – 95% confidence interval of the estimated odds ratio (or beta where reported). Mapped gene – contiguous or adjacent gene mapped to the location of the lead genetic variant. Reported Trait – trait for the GWAS that reported the lead marker from the EBI GWAS catalog. Only “Systemic lupus erythematosus” is included in this table over against, i.e. “lupus nephritis”. Trait(s) – trait or subphenotype. Only “systemic lupus erythematosus” is included in this table over against, i.e. “neonatal lupus”. Background trait – indicator of background trait (i.e. in the case of lupus nephritis in a cohort of SLE patients, SLE would represent the background trait), if present. Study accession – GWAS catalog study identifier. PubMed ID – pubmed ID of the study reporting association. First Author (First author of the study in question). Location – chromosome:position of the lead variant on human genome build 38 (hg38). P – P value converted from format in EBI GWAS catalog to scientific notation. Chromosome – chromosome of lead variant. Position (hg38) position in base pairs of the variant of the lead variant on human genome build 38 (hg38). Region – numerical value of this GWAS region as associated with SLE. Putative Causal gene as predicted by open targets genetics L2G algorithm. Opentargets – link to opentargets genetics prediction and evidence supporting this prediction for that region. “NR” or “‘-” indicates value not reported in the EBI GWAS catalog.

Supplementary Table 2B Human T1D Polygenic risk variants from GWAS catalog and putative causal gene(s) as identified by OpenTargetsGenetics L2G pipeline – columns are identical to **Supplementary Table 2A**, except that they apply to type 1 diabetes and not Systemic lupus erythematosus.

Supplementary Table 3 Genes involved in lupus, type 1 diabetes, peripheral and central B cell tolerance from mouse models – Murine locus – genetic locus, gene name or common protein name of the gene. Gene location – murine chromosome and cytoband of the gene in question. GL Murine – gene location on chromosome in centimorgans. Human orthologue – where identifiable, the human orthlogue to the murine gene in question. Gene location (human) chromosome and cytoband of that human gene. Gene name – HGNC gene name of the gene where applicable. (HGNC = HUGO Gene Nomenclature Committee [https://www.genenames.org/]). Protein – abbreviation or common name for the protein encoded by the gene. Pathway – pathway implicated in the function of this gene in the corresponding class. Model – murine model where gene was implicated. Phenotype – phenotype resulting from gene alteration. Class – murine disease/model state implicated: either lupus, T1D, peripheral tolerance, central tolerance or some combination. PMID – PubMed ID of the publications supporting the link of the gene with a particular class.

Supplementary Table 4 hyperlinks to Networks. This table consists of references to networks in this paper along with links to permanent versions of the networks and analyses that were used to develop the conclusions of this paper. Disease network – disease network as referred to in this paper. Color – color encoding of the disease network in question in –. Shape – shape encoding of the disease network in question in –. URL (NDEX) – universal resource locator for network @ http://www.ndexbio.org URL(string-db.org) – universal resource locator for network @ https://www.string-db.org/URL (Enrichr) – universal resource locator for network @ Enrichr pathway and geneset enrichment analysis: https://maayanlab.cloud/Enrichr/.
