# Supplementary material for: Polygenic autoimmune disease risk alleles impacting B cell tolerance act in concert across shared molecular networks in mouse and in humans
Source: Front Immunol. 2022 Aug 24;13:953439. doi: 10.3389/fimmu.2022.953439 (PMC9450536; doi:10.3389/fimmu.2022.953439)
Supplement: Supplementary file 1 [file DataSheet_1.pdf]

Supplemental Note:

Method for synthesizing published GWAS results as a putative polygenic causal gene network

Detailed step-by-step instructions and graphical overview can be found at the following site:
https://github.com/harleyi/gwas_catalog2polygenic_risk_network

Part 1: make a list of the published genome-wide significant regions from the EBI GWAS catalog

1. Go to EBI/NHGRI GWAS catalog: [https://www.ebi.ac.uk/gwas/]
2. Search your trait of interest
3. Scroll down to associations section and click "export data" button
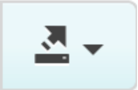
4. Click on CSV and open resulting file in excel
5. sort by trait
6. Remove any traits that are not of interest (i.e. lupus nephritis, neonatal lupus)
7. Sort location by chromosome & position
8. Remove markers that lack mapping information in the EBI GWAS catalog.
9. Remove variants with P-value > 5E-8
10. Define regions. Starting with first variant row, go through all of the rows and increase the region number each time a marker that is a genome-wide significant (P < 5 E-8) marker > 250 000 bases away is observed or a marker on a different chromosome is observed.

Part 2: collate the putative causal genes for each region from the published open targets genetics L2G pipeline results

1. Search the open targets genetics website for trait of interest [https://genetics.opentargets.org]
2. For each GWAS study of the trait of interest in open targets genetics and each region in the table from part 1, add the gene listed in L2G column ("Genes prioritised by our locus-to-gene model with score ≥ 0.5") to the "Putative Causal Gene" column

Part 3: enter the putative causal genes into string-db.org and to putative polygenic causal gene network

1. Go to string-db.org
2. Click search
3. Click multiple proteins on the left hand side
4. Remove commas and ensure that each putative causal gene is on a single line
5. Enter this list of genes in the "list of names" box
6. Select Homo sapiens as organism

7. Search
8. Review gene names and descriptions to ensure that the correct mapping occurred.
9. Click continue
10. To merge networks.
    a. open network of interest in cytoscape
    b. click exports and send network to cytoscape
    c. click merge networks in cytoscape

(OPTIONAL) Part 4: Calculate overlap between two gene networks using hypergeometric distribution

– You can then use these numbers to calculate whether the gene overlap is greater than expected by chance between networks of interest using the cumulative distribution function of the hypergeometric distribution.

A simple web-based calculator can be found at:
https://systems.crump.ucla.edu/hypergeometric/index.php

For an example, we will take two networks, a first network with 54 nodes and a second network with 127 nodes.

The Union of these two networks is a gene network with 172 nodes and the intersection 9 nodes.

Since there are 19566 distinct genes in the current release (v11.5) of the STRING interaction network for *Homo sapiens*, the calculation can be setup as follows:

– *k = 9* (the intersection of the two disease networks is the number of successes).
– *s = 54* (the sample size is the number of nodes in the first gene network).
– *M = 127* (the number of successes in the population is the number of nodes in the second gene network).
– *N = 19566-54 = 19439* (the population size is the entire Homo sapiens gene network with the 54 genes in the first network removed).

Output:
```
Parameters: 9, 54, 127, 19439
expected number of successes = 0.352795925716343
the results are over enriched 25.51 fold compared to expectations
hypergeometric p-value = 6.755808180896782e-11
```

References:
[EBI GWAS Catalog]
Buniello A, MacArthur JAL, Cerezo M, Harris LW, Hayhurst J, Malangone C, McMahon A, Morales J, Mountjoy E, Sollis E, Suveges D, Vrousgou O, Whetzel PL, Amode R, Guillen JA, Riat

HS, Trevanion SJ, Hall P, Junkins H, Flicek P, Burdett T, Hindorff LA, Cunningham F and Parkinson H. The NHGRI-EBI GWAS Catalog of published genome-wide association studies, targeted arrays and summary statistics 2019.
Nucleic Acids Research, 2019, Vol. 47 (Database issue): D1005-D1012.
https://www.ebi.ac.uk/gwas/

[EBI GWAS Catalog Manual]
https://www.ebi.ac.uk/gwas/docs

[L2G model]
Mountjoy, E., Schmidt, E.M., Carmona, M. et al. An open approach to systematically prioritize causal variants and genes at all published human GWAS trait-associated loci. Nat Genet 53, 1527–1533 (2021). https://doi.org/10.1038/s41588-021-00945-5

[L2G Overview & Documentation]
https://genetics-docs.opentargets.org/our-approach/prioritising-causal-genes-at-gwas-loci-l2g

[Open Targets Genetics Portal & Documentation]
https://genetics.opentargets.org/
https://genetics-docs.opentargets.org/

[STRING Database]
Szklarczyk D*, Gable AL*, Nastou KC, Lyon D, Kirsch R, Pyysalo S, Doncheva NT, Legeay M, Fang T, Bork P‡, Jensen LJ‡, von Mering C‡.
The STRING database in 2021: customizable protein–protein networks, and functional characterization of user-uploaded gene/measurement sets .
Nucleic Acids Res. 2021 Jan 8;49(D1):D605-12.PubMed

Szklarczyk D, Gable AL, Lyon D, Junge A, Wyder S, Huerta-Cepas J, Simonovic M, Doncheva NT, Morris JH, Bork P‡, Jensen LJ‡, von Mering C‡.
STRING v11: protein-protein association networks with increased coverage, supporting functional discovery in genome-wide experimental datasets.
Nucleic Acids Res. 2019 Jan; 47:D607-613.PubMed

[STRING Database website]
https://string-db.org/

[STRING & Cytoscape tutorial youtube channel from Lars Juhl Jensen]
https://www.youtube.com/c/LarsJuhlJensen

[Cytoscape]
Shannon P, Markiel A, Ozier O, Baliga NS, Wang JT, Ramage D, Amin N, Schwikowski B, Ideker T. Cytoscape: a software environment for integrated models of biomolecular interaction networks

Genome Research 2003 Nov; 13(11):2498-504

[Cytoscape website, documentation and manual]
https://cytoscape.org/what_is_cytoscape.html
https://github.com/cytoscape/cytoscape-tutorials/wiki
https://manual.cytoscape.org/en/stable/

[Web-based hypergeometric distribution calculator]

https://systems.crump.ucla.edu/hypergeometric/index.php

from the Graeber lab:
https://systems.crump.ucla.edu/
